# A Lightweight Continuous Authentication Protocol for the Internet of Things

**DOI:** 10.3390/s18041104

**Published:** 2018-04-05

**Authors:** Yo-Hsuan Chuang, Nai-Wei Lo, Cheng-Ying Yang, Ssu-Wei Tang

**Affiliations:** 1 Department of Information Management, National Taiwan University of Science and Technology, Taipei 10607, Taiwan; D10009103@mail.ntust.edu.tw (Y.-H.C.); nwlo@cs.ntust.edu.tw (N.-W.L.); M10209101@mail.ntust.edu.tw (S.-W.T.); 2 Department of Computer Science, University of Taipei, Taipei 10048, Taiwan

**Keywords:** continuous authentication, device-to-device authentication, token technique, dynamic device feature, Internet of Things

## Abstract

Modern societies are moving toward an information-oriented environment. To gather and utilize information around people’s modern life, tiny devices with all kinds of sensing devices and various sizes of gateways need to be deployed and connected with each other through the Internet or proxy-based wireless sensor networks (WSNs). Within this kind of Internet of Things (IoT) environment, how to authenticate each other between two communicating devices is a fundamental security issue. As a lot of IoT devices are powered by batteries and they need to transmit sensed data periodically, it is necessary for IoT devices to adopt a lightweight authentication protocol to reduce their energy consumption when a device wants to authenticate and transmit data to its targeted peer. In this paper, a lightweight continuous authentication protocol for sensing devices and gateway devices in general IoT environments is introduced. The concept of valid authentication time period is proposed to enhance robustness of authentication between IoT devices. To construct the proposed lightweight continuous authentication protocol, token technique and dynamic features of IoT devices are adopted in order to reach the design goals: the reduction of time consumption for consecutive authentications and energy saving for authenticating devices through by reducing the computation complexity during session establishment of continuous authentication. Security analysis is conducted to evaluate security strength of the proposed protocol. In addition, performance analysis has shown the proposed protocol is a strong competitor among existing protocols for device-to-device authentication in IoT environments.

## 1. Introduction

As megacities have emerged rapidly around the world in recent years, how to support all kinds of activities generated as a result of the huge city populations has become an important and practical issue to government agencies. The visions of smart homes, smart buildings and smart cities are some of the promising solutions possible by generating and utilizing new information from cities themselves to intelligently lift up the support level and range of city governments. To realize this vision, various sensing devices and gateways have to be massively deployed so they can collect different new information through their sensing devices and deliver this information to intelligent backend application systems to produce and support value-added services [1] within city environments. To effectively connect sensing devices, intermediary gateways, backend application servers and client devices such as smartphones, tablets and smart watches [2] for data exchange and information delivery, new infrastructure is required. The concept of the Internet of Things (IoT) [3] indicating the huge number of “things” (objects) which are interconnected through the Internet is suitable to be adopted and defines the new infrastructure as an IoT infrastructure. With deployed IoT infrastructure, a lot of intelligent management services and information synthesis-oriented services can be developed and implemented in various industry sectors such as smart healthcare, intelligent transportation, factory energy management and home appliance control [4,5].

**Figure 1:**
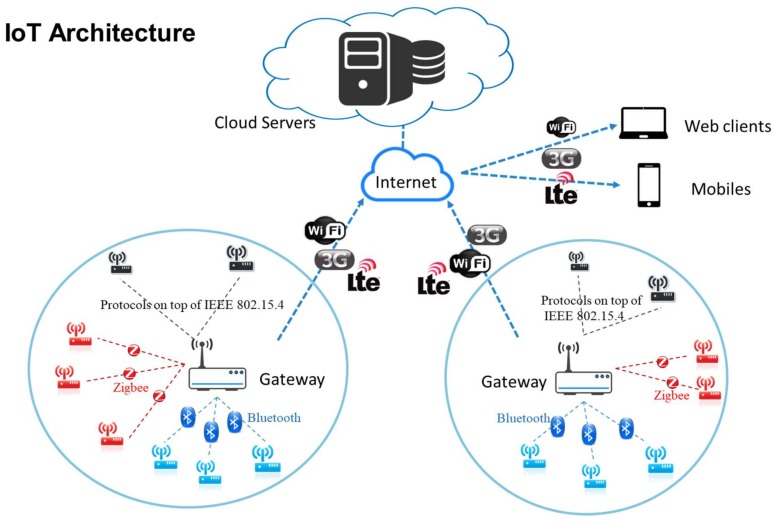
The generic architecture of the Internet of Things.

The development and deployment of IoT infrastructure is in its infancy. The generic architecture of the Internet of Things is shown in Figure 1. The architecture model is composed of fours parts: sensor nodes (devices), gateways, cloud servers, and users. They are interconnected through wired and/or wireless technologies. In general, a gateway connects and manages several corresponding sensing devices [6]. As most of sensing devices deployed in an IoT environment are physically accessible by adversaries, physical security of device hardware and information security for device communications have become serious concerns for the deployment of IoT infrastructure. In addition, heterogeneous hardware components and functionalities along with support complexity on multiple communication protocols among sensing devices and gateways have brought more security challenges to IoT environments. Among security issues [7,8] of IoT environments, such as data protection and access control, device authentication between a sensing device and a gateway is a fundamental and indispensable security feature.

Authentication is an indispensable security mechanism. The goal is to identify the legitimacy of an entity such as a device or a user. Based on previously published literature, user authentication is divided into two categories: static authentication and continuous authentication [9]. A general static user authentication process will be invoked at the beginning of a communicating session to authenticate the identity of a user, who is trying to log into a corresponding service server [9,10]. In general, a secret known, possessed or biologically inherited by the genuine user, such as password, PIN, smartcard, security token, face features, and fingerprint, will be used as the input of an authentication request [11,12]. Unfortunately, static authentication cannot defend against session hijacking attacks. In order to strengthen security, the concept of continuous authentication is developed. Continuous authentication can repeatedly authenticate the legitimacy of a user during the time of device usage. However, continuous authentication is not a substitute for static authentication [12]. In fact, it complements static authentication to enhance security strength. Existing approaches for continuous user authentication employ behavioral biometrics such as mouse and keystroke dynamics to continuously check the authenticity of a user [10,13]. In [14], shared secrets were used to construct authentication tokens; after both communicating parties (the user and the server) agree on the secret used in this authentication session, in a pre-defined time interval only the tokens and corresponding messages are required to be transmitted from the user to the server. The server can verify the received messages are sent from the genuine user based on the associated tokens. In previously published literature, continuous authentication mechanisms were generally applied to user-to-device authentication models [10,13,14].

In an IoT environment, how to accomplish device-to-device authentication has become a practical and fundamental issue. Most sensing devices have limited computing resources and storage capacity [14]. These devices cannot perform complex computations such as encryption and decryption operations unless they have been equipped with a sufficient amount of flash memory and enough computing power microcontrollers (e.g., constrained devices categorized as class 2 in [15,16]). Existing approaches have proposed lightweight protocols to authenticate the legitimacy of a peer device when a message needs to transmit to the peer device [8,17,18,19,20,21]. However, it is possible that a lot of instantaneous messages are transmitted in a short time period between a sensor node and a gateway in an IoT environment. Under such circumstances, adopting existing device-to-device authentication solutions may consume a lot of time in the authentication process in comparison with the time required for processing the received message. Therefore, the goal of this study is to design a lightweight device-to-device continuous authentication protocol to authenticate each data message exchanged between two devices within a pre-defined time period under IoT environment conditions.

The proposed lightweight continuous authentication protocol has the following features: (i) the protocol supports mutual authentication, i.e., both peer devices can authenticate each other before transmitting any messages; (ii) the protocol only uses lightweight computation operations, which include hash-based message authentication (HMAC) [22], hash function, and bitwise exclusive-or (XOR) operation, such that most sensing devices with limited computing resource have a good chance to adopt this protocol and install the corresponding protocol module; (iii) the proposed protocol contains two phases: the static authentication phase dynamically generates an agreed initial token for both communicating parties and the continuous authentication phase transmits authenticated initial token along with sensed data from the sensor device to the gateway. The protocol adopts token technique to support continuous authentication in which the session token secretly contains the dynamic (or time-dependent) feature of the sensing device, i.e., the remaining battery capacity of the sensing device in the proposed scheme. In addition, the time-bounded concept of valid authentication time period is proposed to enhance the security robustness of authentication between IoT devices. Security analysis and performance analysis are conducted to evaluate security strength and time consumption of the proposed protocol. The proposed protocol can also be extended in two aspects: adopting the protocol implementation option of gateway-initialized request and adding the feature of identity anonymity onto sensing devices.

The rest of this paper is organized as follows: related literature surveys on IoT authentication and continuous authentication are addressed in Section 2. In Section 3, the proposed lightweight authentication protocol is depicted. In Section 4, security and performance analyses for the proposed protocol are presented. Section 5 presents possible extensions of the proposed protocol. The final conclusions are addressed in Section 6.

## 2. Related Work

As IoT environments are open or semi-open to their potential users in general, adversaries can easily access devices deployed within an IoT environment. Therefore, IoT environments are vulnerable to various security threats. In consequence, authentication mechanisms should be implemented to provide secure communication between devices. In the section, related literature on traditional authentication and continuous authentication for IoT environments are discussed.

### 2.1. IoT Authentication

In this subsection, we categorize existing device-to-device authentication protocols for IoT environments into three groups: certification-based authentication, encryption-based authentication, and non-encryption-based authentication.

- *Certification-based Authentication*: The Datagram Transport Layer Security (DTLS) [23] protocol is an existing standard. In 2013, Kothmayr et al. [24] proposed a two-way authentication security scheme for IoT based on DTLS, which used RSA-based asymmetric encryption and X.509 certification. However, this scheme needs eight handshakes to establish a session. Hence, in order to implement this scheme, higher consumption cost and more storage space are required from resource-constrained sensing devices. In 2014, Porambage et al. [21] proposed an authentication protocol by using implicit certificate in distributed IoT environments. Since Elliptic Curve Cryptography (ECC) consumes less computing resources relative to RSA, the protocol in [21] employs the Elliptic Curve Qu-Vanstone (ECQV) implicit certificate scheme and the Elliptic Curve Diffie-Hellman (ECDH) key exchange scheme. The protocol uses implicit certificates to accomplish end-to-end authentication in distributed IoT environments. The protocol contains two authentication phases, which are the registration phase and the authentication phase. Although the proposed scheme adopts ECC to reduce computation overhead for sensing devices, the protocol still requires some storage space in devices to store implicit certificates and the scheme also requires a Certificate Authority (CA).
- *Encryption-based Authentication*: In 2015, Shivraj et al. [8] proposed One Time Password (OTP) authentication for IoT infrastructures. The protocol adopts Identity Based Elliptic Curve Cryptography (IBE-ECC) to provide a lightweight end-to-end authentication between devices. The advantage of the protocol is that sensing devices do not need extra storage for storing the keys as the scheme uses OTP. However, if the devices need to communicate frequently, they must frequently request the central cloud to generate the OTP. In consequence, communicating devices may spend more time to establish a session. In 2015, Mahalle et al. [20] proposed a group authentication protocol for IoT environments. The protocol could effectively authenticate the devices in the same group. The proposed TCGA scheme uses Paillier Threshold Cryptography. The Paillier Cryptosystem possesses special properties such as homomorphic addition, indistinguishability, and self-binding [25,26]. The TCGA scheme establishes a session key for each group authentication to achieve efficient authentication among group members. The main concern for this scheme is that if a new device member joins the group, the keys for group members have to be regenerated and distributed to all members again. If the targeted IoT environment needs to frequently change device members in their group, it may cause additional authentication overhead for the devices. In 2015, Khemissa and Tandjaoui [18] proposed a lightweight authentication for IoT environments without using complex cryptographic operations. The protocol employed hash-based message authentication code (HMAC) [22] operations and nonce to establish mutual authentication. Advanced Encryption Standard (AES) [27] encryption mechanism was used to generate a session key. Hence, the scheme requires sensing devices to possess the ability to perform symmetric cryptographic operations. In 2016, Khemissa and Tandjaoui [19] extended their work in [18] to support remote users. The protocol could achieve mutual authentication between a sensor node and a remote user. A user could use his/her mobile device to manage heterogeneous sensing resources. In 2016, Kumar et al. [28] presented a lightweight authentication-based session key establishment protocol for smart home. The protocol requires a security service provider, which is a trusted server. The security service provider assigns important parameters, generates tokens and distributes tokens to communicating devices in a smart home environment. The devices use authenticated token to establish a session key and achieve mutual authentication.
- *Non-encryption-based Authentication*: In this category, proposed approaches do not use any certification technique or any encryption operation. In 2015, Gope et al. [29] proposed an untraceable authentication protocol in distributed IoT architecture. The scheme only uses hash functions and bitwise exclusive-or operations to construct a lightweight authentication mechanism. In addition, the scheme uses sequence numbers and random numbers to generate a one-time alias identity. The proposed scheme not only ensures the legality of a sensor node but also support identity anonymity and untraceability. In 2015, Kawamoto [30] presented a location-based authentication scheme in IoT environments. The protocol utilizes ambient information of devices for authentication. The scheme has to continuously collect ambient information from IoT devices.

### 2.2. Continuous Authentication

In this subsection, we review related literature on continuous authentication. We classify continuous authentication protocols into two categories: user-to-device models and device-to-device models:

- *User-to-Device Models*: Several schemes for continuous user authentication have been proposed in recent years [10,31,32,33,34,35]. The goal of these proposed solutions are to help devices to constantly authenticate the current user to prevent impersonated or illegal users using devices. The communication model of these schemes is user-to-device and most schemes utilize behavioral biometrics to construct their continuous authentication process. In 2010, Shimshon et al. [31] presented a continuous authentication mechanism which repeatedly verifies the identity of current device user based on keystroke dynamics. The proposed scheme collects multiple keystrokes from the genuine user to create corresponding feature vectors and use these vectors as the reference base. Once a genuine user gets authenticated to use the device with continuous authentication module, within pre-defined time period the module will repeatedly collect newly generated keystrokes, generate corresponding feature vectors and compare them with the reference base in order to validate the current user is indeed the one authenticated. In 2012, Shen et al. [32] proposed a continuous authentication protocol based on dynamic patterns of mouse usage by a genuine user. There are other approaches adopting multi-behavioral biometrics to construct continuous authentication mechanism. In 2014, Bailey et al. [33] proposed a continuous user authentication scheme using the combined patterns of keyboard, mouse, and Graphical User Interface (GUI) interactions generated from a user as the reference base of a specific genuine user to achieve higher authentication accuracy. In 2015, Buduru and Yau [10] introduced a continuous user authentication scheme based on the patterns of user finger gestures on the touch screen of a targeted device. Modified Markov Decision Process (MDP) models for different usage contexts are adopted by the scheme of Buduru and Yau. In 2010, Niinuma et al. [34] adopted soft biometric features including facial skin and clothing color to construct their continuous user authentication mechanism. In 2012, Mock et al. [35] proposed their continuous user authentication scheme based on a user iris recognition mechanism. This scheme could also add user password option to establish a multi-factor user authentication solution. In 2017, Peng et al. [36] introduced a continuous authentication mechanism for users who wear smart glasses to protect user privacy. This mechanism utilizes finger touch gestures and voice commands to construct their behavioral biometrics. In 2017, Zhou et al. [37] proposed a transparent authentication scheme to continuously authenticate targeted user through authentication token, which contains the brainwave patterns of the targeted user.
- *Device-to-Device Models*: To the best of our knowledge, there are no studies on device-to-device continuous authentication in IoT environments. In 2015, Bamasag and Youcef-Toumi [14] proposed a lightweight continuous user authentication for IoT environments. Their work identified the need of continuous authentication in IoT environments. As sensing devices in particular scenarios, such as personal health monitoring and industrial control systems [38], need to frequently transmit sensed data to gateways in a short period of time, a continuous authentication mechanism could accomplish faster authentication. In the proposed scheme, secret shares are used to construct authentication tokens and only the tokens and corresponding messages are required to be transmitted from the user to the server in a pre-defined time interval for continuous authentication. The server can verify the received messages are sent from the genuine user based on the associated tokens. Even though the proposed protocol in [14] is under user-to-device model, it inspires us in many aspects to design a new lightweight device-to-device continuous authentication protocol.

## 3. The Proposed Scheme

In this section, we introduce the proposed authentication protocol. We will describe our design concept, assumptions, and notations of our proposed protocol. The proposed protocol consists of three phases: initialization phase, static authentication phase, and continuous authentication phase.

### 3.1. Design Concept

In some IoT environments such as factory monitoring and smart inpatient systems, sensor nodes frequently transmit a large number of sensed data to the gateway in a short time period. Since the time interval of each data transmission session is very short, the gateway must frequently authenticate the communicating devices (sensor nodes) in the beginning of each data transmission session. In order to fast ensure authenticity of devices for each received data in a valid session by a gateway, we adopt the design of continuous authentication. Continuous authentication can save authentication time for each data transmission session. The proposed protocol utilizes the value of remaining battery capacity as a dynamic factor to authenticate the targeted sensing device.

**Figure 2:**
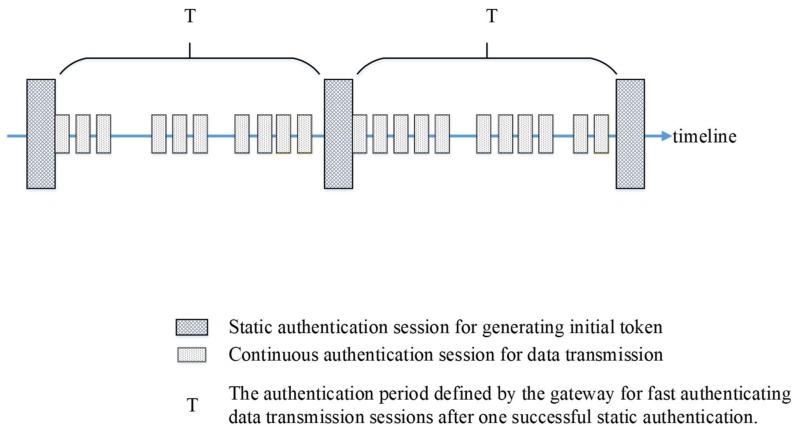
The proposed authentication protocol framework through timeline.

The proposed authentication protocol contains two authentication phases, which are the static authentication phase and the continuous authentication phase. In each phase, we have developed a corresponding authentication scheme. Static authentication scheme is similar to general or traditional authentication approaches and it is applied to authenticate devices in the beginning of an authentication period *T*. Continuous authentication scheme is applied to each sensed data transmission during the current authentication period *T*. To further clarify the proposed mechanism, Figure 2 shows the protocol framework through timeline, in which dot blocks indicate static authentication sessions and reticular blocks indicate continuous authentication sessions. If a sensor node transmits sensed data to a gateway, the sensor node and the gateway mutually authenticate each other during the static authentication phase. After passing the static authentication phase, the continuous authentication scheme is applied to each sensed data transmission from the sensor node to the gateway through the current authentication period *T*. There will be some time intervals in which no data transmission occurs during an authentication period *T*.

Within the pre-defined authentication period *T*, the static authentication process is first invoked for communicating devices to set up an authenticated token, which will be used for each continuous authentication session. Then, during the authentication period *T*, the gateway can quickly verify the legality of the sensor node when a new message or data needs to be transmitted between both parties. With the use of an authenticated token, the continuous authentication scheme spends less time for computation than the static authentication scheme. The proposed protocol does not utilize any cryptographic operation; therefore, it is a lightweight authentication protocol.

**Figure 3:**
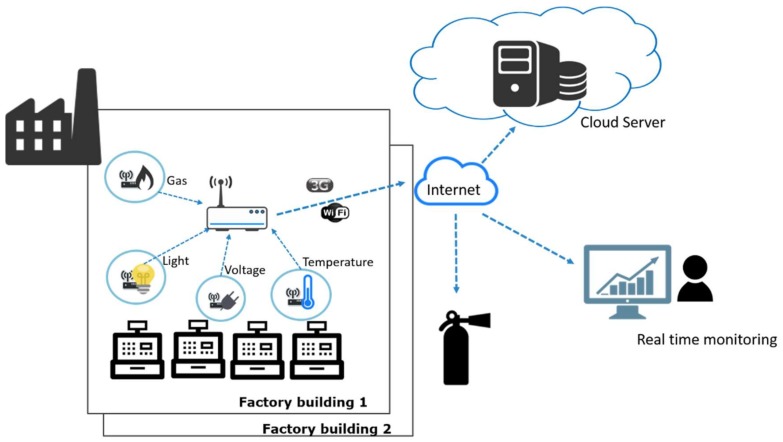
The possible IoT architecture for factory monitoring scenario.

Next, we introduce two general scenarios in IoT environments to show the demand of lightweight device-to-device continuous authentication. The first scenario is factory monitoring as shown in Figure 3. For a factory monitoring system to be well functioned, many different types of sensing devices and intermediary gateways need to be deployed in a factory building. Each gateway can manage a number of sensing devices. Sensor nodes (devices) can obtain various sensed data such as machinery voltage, equipment vibration, machine temperature and pressure, and environmental information like humidity and temperature. The sensed data are collected by sensing devices and later transmitted to a gateway. After the gateway has received the data, it transmits sensed data to a cloud server or a local backend server. The sensed data are used to monitor the real-time production status of the factory. If there are abnormal situations occurred, corresponding safety alarms and warning messages will be generated immediately by the monitoring system. Since the system needs to gather real-time sensed data constantly, the sensing devices have to transmit their sensed data to intermediary gateways frequently in a short time period.

**Figure 4:**
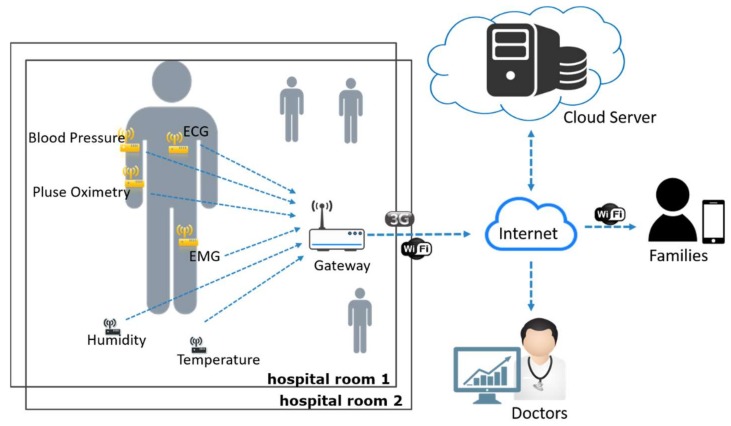
The possible IoT architecture for smart inpatient system scenario.

The other scenario is the smart inpatient system as shown in Figure 4. To implement a smart inpatient system, the hospital needs to install and deploy various sensing devices in a ward or even asks the patients to wear some body sensors. These sensor nodes can acquire sensed data such as the patient’s temperature, ECG, and blood pressure, and other environmental information. Similar to the first scenario, all sensed data will be collected to a cloud server or a local server. Doctors and nurses can watch over real-time body status of all inpatients and the environmental information of each ward through the system dashboard. In this scenario, the sensed data also needs to be transmitted periodically to the backend server in a short time period.

### 3.2. Assumptions

We list our assumptions for the proposed protocol as follows:

1. Sensor nodes are resource-limited devices powered by one or more batteries, which have low computational capability and storage space. Each sensor node is able to perform hash operation and has a random number generator to generate random numbers.
2. Gateways are resource-unlimited devices, which have sufficient computational capability to perform hash operation and generate random numbers, and storage space to store temporary values and pre-defined data tables.
3. Multiple sensor nodes can be managed by one gateway. Each sensor node and the corresponding gateway share one distinct secret value which is set in the initialization phase of the proposed authentication protocol.
4. The sensor node cannot precisely digitize and display its remaining energy (or battery) capacity on its display panel (if it has one).

### 3.3. Notations

The notations used are defined in Table 1.

### 3.4. Battery Consumption

- *Estimated Daily Average Battery Consumption (EBC_SN_)*: In order to calculate the estimated daily average battery consumption value for a sensor node, we propose a daily battery consumption equation based on the battery life time and battery capacity of a sensor node:
  (1) $$EBC_{SN}=\frac{BC}{BL}$$
  In Equation (1), $\;EBC_{SN}$ indicates estimated daily average battery consumption value for a sensor node in the unit of milliampere-hour (mAh) per day. BC is the fully charged battery capacity of a sensor node in the unit of mAh. BL is the battery life time of a sensor node in the unit of day. We use battery life time and battery capacity (mAh) of a sensor node to calculate $EBC_{SN}$ whose measurement unit is by mAh/day.
- *Estimated Remaining Battery Capacity Threshold (*$BCT_{SN}$*)*: In order to check the remaining battery capacity within a reasonable value for a sensor node during an authentication period *T*, we design an equation to compute the estimated remaining battery capacity threshold as shown in Equation (2):
  (2) $$BCT_{SN}=\left[rb-\left(\frac{EBC_{SN}}{24\times 60}\times T\right)\;\right]\times s$$
  In Equation (2), $BCT_{SN}$ indicates the estimated remaining battery capacity threshold of a sensor node and the measurement unit of $BCT_{SN}$ is mAh. $EBC_{SN}$ indicates the estimated daily average battery consumption value for a sensor node in the unit of mAh per day. rb is the current energy capacity of a sensor battery. $\left(\frac{EBC_{SN}}{24\times 60}\right)$ indicates the estimated battery consumption value per minute for the sensor node.$\;\left(\frac{EBC_{SN}}{24\times 60}\times T\right)$ indicates the estimated battery consumption value per authentication period T for the sensor node. For simplicity, we assume sensor battery consumption is a linear relationship in association with the running time of a sensor node. Symbol s indicates an estimation coefficient to accommodate possible deviation on the calculated threshold value $\left[rb-\left(\frac{EBC_{SN}}{24\times 60}\times T\right)\;\right]$. The calculated threshold value is multiplied by the coefficient s to form the final value of $BCT_{SN}$. In general, the battery consumption model along with the value of the coefficient s of a senor device could be evaluated and revealed by the corresponding sensor vendor, where 0<s<1.

### 3.5. The Proposed Authentication Protocol

This subsection introduces the details of our proposed protocol, which consists of three phases: initialization phase, static authentication phase, and continuous authentication phase.

#### 3.5.1. Initialization Phase

In the Initialization phase, important parameters have to be set up for both sensor nodes and gateways. First, the sensor node submits its identity $ID_{SN}$ and related battery information which includes battery life time and battery capacity to the gateway via a secure channel. There are many ways to deliver required secret values to both sensors and gateways; for example, sensor vendors can pre-install secret value into sensors during production phase and a gateway can acquire required secret values from a trusted third party server (a broker website or the Web service of targeted sensor vendor). If sensors are class 2 type devices as defined in [15], it is possible for a gateway to establish a secure channel with these sensors through encryption operations.

After the gateway receives the request from a sensor node, the gateway generates a secret value $SK_{SN}$. Subsequently, the gateway also computes the estimated daily average battery consumption value $EBC_{SN}$ for a sensor node SN. Then, the gateway sets an authentication period which is defined by the gateway for fast authenticating data transmission sessions after one successful static authentication. Next, the gateway securely sends the secret value $SK_{SN}$ back to the sensor node via a secure channel. As soon as the sensor node obtains the secret value $SK_{SN}$ from the gateway, it stores the secret value $SK_{SN}$ in its secure storage. Next, the gateway also records the sensor node’s secret value $SK_{SN}$, authentication period *T*, and estimated daily average battery consumption value $EBC_{SN}$ for a sensor node SN into its mapping table or database. Therefore, the gateway stores each managed sensor node’s important information in the binding table whose field contains the identity $ID_{SN}$, the corresponding secret value $SK_{SN}$, authentication period T, and estimated daily average battery consumption value $EBC_{SN}$ for the sensor node SN.

#### 3.5.2. Static Authentication

In the static authentication phase, a sensor node and a gateway mutually authenticate each other within static authentication. Simultaneously, both sides negotiate an initial token $TK_{SN}^{I}$ to be used for continuous authentication during the authentication period 𝑇. The gateway calculates estimated remaining battery capacity threshold $BCT_{SN}$ which is used to check whether the remaining battery capacity value of a sensor node is reasonable when the gateway receives data sent from the sensor node in a data transmission session.

**Figure 5:**
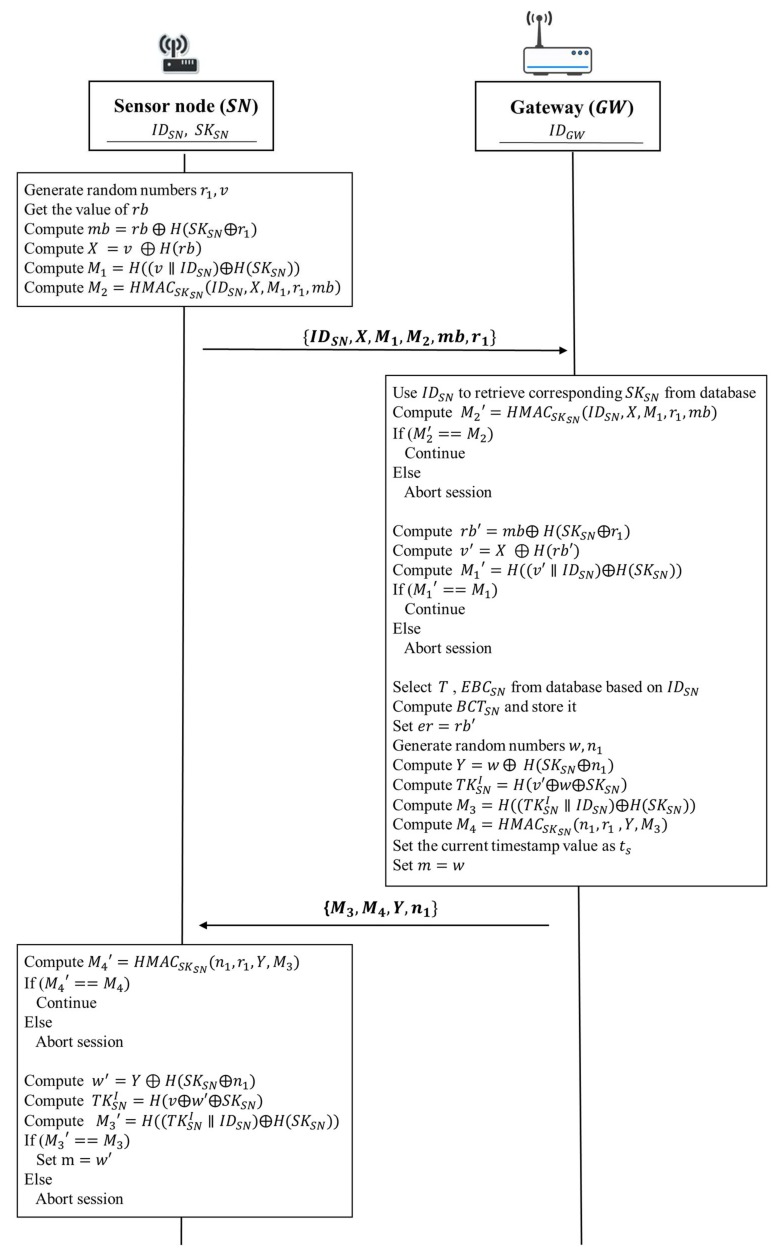
The static authentication phase of the proposed protocol.

The static authentication phase of our proposed protocol is according to the following steps shown in Figure 5:

1. Sensor node → Gateway: $ID_{SN}$, X, $M_{1}$, $M_{2}$, mb, $r_{1}$

A sensor node generates random numbers $r_{1}$ and v. Then the sensor node gets the value of the current energy capacity of sensor battery rb and gets its secret value $SK_{SN}$ from its secure storage. Next, it takes the current energy capacity of sensor battery rb to compute $mb=rb⨁H\left(SK_{SN}⨁r_{1}\right)$. After that, the sensor node computes X=v ⨁ H(rb) and $M_{1}=H\left(\left(v∥ID_{SN}\right)⨁H\left(SK_{SN}\right)\right)$, respectively. Subsequently, the sensor node uses the secret value $SK_{SN}$ to compute $M_{2}=HMAC_{SK_{SN}}\left(ID_{SN},X,M_{1},r_{1},mb\right)$ in order to guarantee that the message will not be modified during data transmission. Finally, the sensor node sends $ID_{SN}$, X, $M_{1}$, $M_{2}$, mb, and $r_{1}$ to the gateway.

2. Gateway → Sensor node: $M_{3}$, $M_{4}$, Y, $n_{1}$

Upon receiving $ID_{SN}$, X, $M_{1}$, $M_{2}$, mb, and $r_{1}$ from the sensor node, the gateway uses the identity of a sensor node $ID_{SN}$ to retrieve corresponding secret value $SK_{SN}$ from its database. Then the gateway uses the secret value $SK_{SN}$ to compute $M_{2}^{\prime }=HMAC_{SK_{SN}}\left(ID_{SN},X,M_{1},r_{1},mb\right)$. After that, the gateway verifies whether the computed value $M_{2}\prime$ is equivalent to the received value $M_{2}$. If $M_{2}\prime$ and $M_{2}$ are equivalent, it indicates that the obtained messages are not changed by any malicious attacker. Otherwise, the gateway will terminate the protocol. Next, the gateway computes $rb^{\prime }=mb⨁H\left(SK_{SN}⨁r_{1}\right)$ and uses $rb^{\prime }$ to compute $v^{\prime }=X\;⨁H\left(rb\prime \right)$. After computing $rb^{\prime }$ and $v^{\prime }$, the gateway takes the random number $v^{\prime }$ to compute $M_{1}{}^{\prime }=H\left(\left(v\prime ∥ID_{SN}\right)⨁H\left(SK_{SN}\right)\right)$. Then, the gateway verifies whether the computed value $M_{1}{}^{\prime }$ is equivalent to the received value $M_{1}$. If $M_{1}{}^{\prime }$ and $M_{1}$ are equivalent, it indicates that the obtained values of v′ and rb′ are correct. Otherwise, the gateway will also terminate the protocol.

After the above verification tasks have been completed, the gateway retrieves the corresponding authentication period T and the estimated daily average battery consumption value $EBC_{SN}$ from its database associated with $ID_{SN}$. Subsequently, the gateway uses the authentication period T and the estimated daily average battery consumption value $EBC_{SN}$ to compute the value of the estimated remaining battery capacity threshold $BCT_{SN}$ for the sensor node. Then the gateway sets er=rb′. After that, the gateway generates random numbers w and $n_{1}$. Then, the gateway performs the two computations to compute $Y=w⨁H\left(SK_{SN}⨁n_{1}\right)$, $TK_{SN}^{I}=H\left(v\prime ⨁w⨁SK_{SN}\right)$, respectively. Next, the gateway uses the initial token $TK_{SN}^{I}$ to compute $M_{3}=H\left(\left(TK_{SN}^{I}∥ID_{SN}\right)⨁H\left(SK_{SN}\right)\right)$. Subsequently, the gateway uses the secret value $SK_{SN}$ and the random number $n_{1}$ to compute $M_{4}=HMAC_{SK_{SN}}\left(n_{1},r_{1},Y,M_{3}\right)$. Then the gateway stores $BCT_{SN}$ and $TK_{SN}^{I}$ in its database. It also sets current timestamp value as $t_{s}$ and sets m=w. Finally, the gateway sends $M_{3}$, $M_{4}$, Y, and $n_{1}$ to the sensor node.

Upon receiving $M_{3}$, $M_{4}$, Y, and $n_{1}$ from the gateway, the sensor node uses the secret value $SK_{SN}$ to compute $M_{4}\prime =HMAC_{SK_{SN}}\left(n_{1},r_{1},Y,M_{3}\right)$. After the computation, the gateway verifies whether the computed value $M_{4}\prime$ is equivalent to the received value $M_{4}$. If $M_{4}\prime$ and $M_{4}$ are equivalent, it indicates that all the obtained messages are not changed. Otherwise, the sensor node will terminate the protocol. Then the sensor node computes $w^{\prime }=$ $Y⨁H\left(SK_{SN}⨁n_{1}\right)$. Next, the sensor node uses the obtained random number $w^{\prime }$ to compute $TK_{SN}^{I}=H\left(v⨁w\prime ⨁\;SK_{SN}\right)$. After that, the sensor node uses the computed initial token $TK_{SN}^{I}$ to compute $M_{3}\prime =H\left(\left(TK_{SN}^{I}∥ID_{SN}\right)⨁H\left(SK_{SN}\right)\right)$. Then, the sensor node verifies whether the computed value $M_{3}{}^{\prime }$ is equivalent to the received value $M_{3}$. If $M_{3}\prime$ and $M_{3}$ are the same, it indicates that the computed initial token $TK_{SN}^{I}$ is correct and authentication task is successful. The sensor node sets m=w′ and stores the initial token $TK_{SN}^{I}$ in the secure storage of the sensor node. Then, the sensor node can perform the continuous authentication for each data transmission during the current authentication period T. Otherwise, the sensor node will terminate the protocol.

#### 3.5.3. Continuous Authentication

Continuous authentication is applied to sensed data transmission from the sensor node to the gateway after static authentication between them has been validated during the current authentication period 𝑇. Since continuous authentication happens after one successful static authentication, the sensor node has stored initial token $TK_{SN}^{I}$, and estimated remaining battery capacity threshold $BCT_{SN}$ for the current authentication period T. After receiving data from a sensor node, the gateway performs a series of verification tasks to ensure the authenticity of a sensor node. First, the gateway checks whether the received message is generated in current authentication period 𝑇. Second, the gateway verifies the value $M_{5\;}$ which indicates data integrity of the transmitted message and the remaining battery capacity 𝑟𝑏 is within a reasonable range. Finally, the gateway sends an acknowledgement ACK to the sensor node.

**Figure 6:**
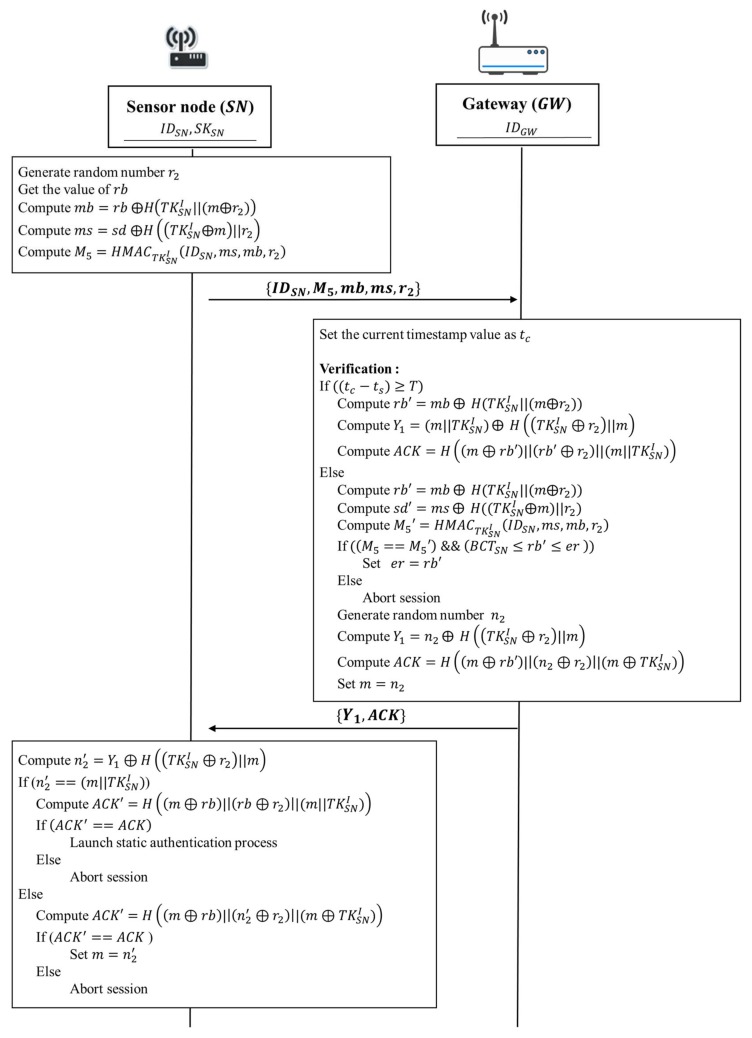
The continuous authentication phase of the proposed protocol.

The continuous authentication phase of our proposed protocol is according to the following steps shown in Figure 6:

1. Sensor node → Gateway: $ID_{SN}$, $M_{5}$, mb, ms, $r_{2}$

Sensor node generates a random numbers $r_{2}$ and gets the value of the current energy capacity of the sensor battery rb. Then, the sensor node gets the initial token $TK_{SN}^{I}$ from its secure storage and takes the current energy capacity of sensor battery rb to compute $mb=rb\;⨁H\left(TK_{SN}^{I}||\left(m⨁r_{2}\right)\right)$. Next, the sensor node uses the random number m which had been generated at the static authentication phase to compute $ms=sd\;⨁H\left(\left(TK_{SN}^{I}⨁m\right)||r_{2}\right)$ for masking the sensed data 𝑠𝑑. After that, the sensor node computes $M_{5}=HMAC_{TK_{SN}^{I}}\left(ID_{SN},ms,mb,r_{2}\right)$ in order to detect that the message has be modified during the data transmission. Next, the sensor node sends $ID_{SN}$, $M_{5}$, mb, ms, and $r_{2}$ to the gateway.

2. Gateway → Sensor node: $Y_{1}$, ACK

Upon receiving $ID_{SN}$,$\;M_{5}$, mb, ms, and $r_{2}$ from the sensor node, the gateway performs a series of verification tasks. First, the gateway sets the current timestamp value as $t_{c}$. Then, the gateway verifies whether the received message is generated in the current authentication period T. If (($t_{c}-t_{s})\geq T)$, it indicates that the timestamp $t_{c}$ is out of range of the period time T. That is, the sensor has to launch the static authentication again for security issue. Therefore, the gateway compute $rb\prime =mb⨁\;H(TK_{SN}^{I}||\left(m⨁r_{2}\right))$, $Y_{1}=(m||TK_{SN}^{I})⨁\;H\left(\left(TK_{SN}^{I}⊕r_{2}\right)||m\right)$ and $ACK=H\left(\left(m⊕rb\right)\left|\left|\left(rb⊕r_{2}\right)\right|\right|(m||TK_{SN}^{I})\right)$ for informing the sensor to relaunch the static authentication process. Then, the gateway sends an acknowledgement ACK and $Y_{1}$ to the sensor node.

After verifying timestamp, the gateway verifies data integrity of the message during the data transmission. Based on the received $ID_{SN}$, the gateway gets the corresponding initial token $TK_{SN}^{I}$ from its database. The gateway uses the initial token $TK_{SN}^{I}$ to compute $b^{\prime }=$ $mb\;⨁\;H(TK_{SN}^{I}||\left(m⨁r_{2}\right))$, $sd^{\prime }=ms\;⨁\;H(\left(TK_{SN}^{I}⨁m\right)||r_{2})$ and $M_{5}\prime =HMAC_{TK_{SN}^{I}}\left(ID_{SN},ms,mb,r_{2}\right)$, respectively. Then the gateway verifies whether the computed value $M_{5}{}^{\prime }$ is equivalent to the received value $M_{5}$. If $M_{5}{}^{\prime }$ and $M_{5}$ are equivalent, it indicates that the obtained message is not modified during transmission. In addition, the gateway also verifies whether the remaining battery capacity rb′ is within a reasonable range. If ($BCT_{SN}\leq rb\prime \leq er\;$), it indicates that the current energy capacity of sensor battery rb′ is within a reasonable range and the validity of the sensor node is authenticated. Otherwise, the gateway aborts the session.

After the above verification tasks have been completed, sensor node is authenticated successfully and the gateway can assure the sensed data sd′ which is transmitted by the sensor node is valid. The gateway sets er=rb′. Then the gateway generates a random number $n_{2}$ to compute $Y_{1}=n_{2}⊕H\left(\left(TK_{SN}^{I}⊕r_{2}\right)||m\right)$ and $ACK=H\left(\left(m⊕rb\prime \right)\left|\left|\left(n_{2}⊕r_{2}\right)\right|\right|\left(m⊕TK_{SN}^{I}\right)\right)$. After that, the gateway sets $m=n_{2}$. Finally, the gateway sends an acknowledgement ACK and $Y_{1}$ to the sensor node.

Once the sensor node receives $Y_{1}$ and ACK, the sensor node computes $n_{2}^{\prime }=$ $Y_{1}⊕H\left(\left(TK_{SN}^{I}⊕r_{2}\right)||m\right).$ If the value of $n_{2}^{\prime }$ is equivalent to $\left(m||TK_{SN}^{I}\right)$, it means that this continuous authentication process is expired. That is, the sensor should relaunch the static authentication again. Before launching the static authentication, the sensor has to compute $ACK\prime =H\left(\left(m⊕rb\right)\left|\left|\left(rb⊕r_{2}\right)\right|\right|(m||TK_{SN}^{I})\right)$ to verify data integrity of the received message. If $\left(ACK^{\prime }==ACK\right)$, it indicates that the message have not been modified during the data transmission. Then, the sensor relaunch the static authentication process. Otherwise, the sensor aborts the session.

On the other hand, if the value of $n_{2}^{\prime }$ is not equivalent to $\left(m||TK_{SN}^{I}\right)$, this continuous authentication should be successful. In order to verify data integrity of the received message, the sensor computes $ACK\prime =H\left(\left(m⊕rb\right)\left|\left|\left(n_{2}^{\prime }⊕r_{2}\right)\right|\right|\left(m⊕TK_{SN}^{I}\right)\right)$ to compare with the received value of ACK. If the value of ACK′ is equivalent to ACK, this continuous authentication is successful. Finally, the sensor node sets $m=n\prime_{2}$ and goes to the next continuous authentication for each data transmission. Otherwise, the protocol will be terminated. The sensor node must receive the acknowledgement ACK to indicate a graceful session ending.

## 4. Protocol Analysis

### 4.1. Security Analysis

In this section, a security analysis is conducted to evaluate the security robustness of the proposed protocol. There are six security properties supported by the protocol design: resistance to replay attack, resistance to impersonation attack, resistance to man-in-the-middle attack, data integrity, mutual authentication, and forward secrecy.

- *Resistance to Replay Attacks*: A malicious attacker may eavesdrop valid messages transmitted during an authentication session. Later on, the attacker replays some of these messages to impersonate a legitimate entity for establishing an authenticated session with the target peer. In our proposed protocol, if an attacker eavesdrops messages and performs a replay attack, the message receiver (the gateway or a sensor) can detect those messages are invalid. In our proposed protocol, the values $M_{2}$, $M_{4}$, $M_{5}$, and ACK containing transmitted messages are all constructed with fresh random numbers and each value will be transmitted along with its corresponding random number which is used as one of the variables to dynamically generate the value. These random numbers are freshly generated by both communicating parties during each authentication session. The message receiver will verify the validity of the received message by using the received random number to generate a tentative message and evaluating the equivalence between the received message and the tentative one. If both messages are equivalent, then the message receiver determines the received message is valid. As our protocol embeds random numbers into individual messages to keep freshness of the transmitting messages, our proposed protocol is able to resist any replay attack.
- *Resistance to Impersonation Attacks*: An impersonation attack indicates that a malicious attacker may try to masquerade as a valid sensor node. In the static authentication phase, if an attacker wants to masquerade as a sensor node, the attacker will need to forge the message {$ID_{SN},\;X,M_{1},M_{2},\;mb,r_{1}$} sent to the gateway. If an attacker wants to forge the value $M_{2}$, the attacker needs to learn the secret value $SK_{SN}$. The attacker may know the sensor identity $ID_{SN}$ from the eavesdropped messages, but it is unable to learn the secret value $SK_{SN}$. Without knowing the secret value $SK_{SN}$, the attacker cannot compute a valid $M_{2}$. Therefore, the impersonation attack will fail. In the continuous authentication phase, if an attacker wants to masquerade as a valid sensor node, the attacker will need to forge the message {$ID_{SN},M_{5},mb,ms,r_{2}$} sent to the gateway. Therefore, the attacker needs to forge the value $M_{5}$. In consequence, the attacker needs to know the initial token $TK_{SN}^{I}$, the random numbers $r_{2}$ and m, and the current energy capacity of sensor battery rb. The attacker may learn the random numbers $r_{2}$ from eavesdropped messages but it cannot learn the initial token $TK_{SN}^{I}$ from eavesdropped messages. Hence, the attacker cannot compute a valid initial token value $TK_{SN}^{I}$ without knowing the value of the secret value $SK_{SN}$. In summary, the attacker cannot masquerade as a valid sensor node successfully. Hence, our protocol can resist the impersonation attack.
- *Resistance to Man-in-the-middle Attacks*: A man-in-the-middle attack indicates that an active attacker secretly relays and manipulates the messages transmitted between two parties who believe they are directly communicating with each other. In our static authentication phase, if a malicious attacker wants to relay and manipulate transmitting messages, the attacker needs to learn the secret value $SK_{SN}$ and the remaining energy capacity of sensor battery rb. Since the attacker cannot know the secret value $SK_{SN}$ and the remaining energy capacity of sensor battery rb from previously eavesdropped messages, the attacker cannot learn the authentic data and manipulate messages successfully. In the continuous authentication phase, if the malicious attacker wants to relay and manipulate transmitting messages, the attacker needs to obtain the initial token $TK_{SN}^{I}$. As the initial token $TK_{SN}^{I}$ is generated and securely sent in the static authentication phase, the attacker cannot learn the initial token $TK_{SN}^{I}$. The attacker can only eavesdrop the values of mb and ms, but it still cannot learn the authentic data rb and sd which are carefully hidden in mb and ms, accordingly. In consequence, the attacker cannot modify or manipulate transmitting messages without knowing the initial token $TK_{SN}^{I}$. Therefore, the proposed protocol can resist man-in-the-middle attacks.
- *Data Integrity*: Data integrity indicates that a message receiver can ensure the message is not tampered with during transmission. Our protocol adopts an HMAC function to ensure data integrity. In our static authentication phase, if an attacker tries to tamper transmitting messages, the attacker needs to learn the secret value $SK_{SN}$. Since the attacker cannot learn the secret value $SK_{SN}$ from eavesdropped messages, he cannot compute valid values $M_{2}$ and $M_{4}$ without knowing the secret value $SK_{SN}$. Hence, a malicious attacker cannot tamper transmitting messages successfully. In the continuous authentication phase, if an attacker tries to tamper transmitting messages, the attacker needs to learn the initial token $TK_{SN}^{I}$. As the attacker cannot learn the initial token $TK_{SN}^{I}$ from eavesdropped messages, he cannot compute a valid value $M_{5}$ without knowing the initial token $TK_{SN}^{I}$. Hence, our proposed protocol achieves the data integrity property.
- *Mutual Authentication*: Mutual authentication indicates that two entities can authenticate each other. In the static authentication phase, the gateway authenticates the sensor node by verifying the value $M_{2}$ with the shared secret value $SK_{SN}$. If the computed value $M_{2}\prime$ is equivalent to the received value $M_{2}$, the gateway will be able to ensure the validity of the sensor node. Next, the sensor node also authenticates the gateway by verifying the value $M_{4}$ with the shared secret value $SK_{SN}$. The value $M_{4}$ embeds the random numbers $r_{1}$ and $n_{1}$. If the computed value $M_{4}\prime$ is equivalent to the received value $M_{4}$, the sensor node ensures the validity of the gateway. In the continuous authentication phase, both sensor and gateway can authenticate each other via initial token $TK_{SN}^{I}$ and transmitted random numbers. First, the gateway authenticates the sensor node by verifying the value of $M_{5}$ which is encrypted by the initial token $TK_{SN}^{I}$. If the value of $M\prime_{5}$ is equivalent to $M_{5}$, the gateway ensures that the sensor node is valid. Next, the sensor node authenticates the gateway by verifying the value ACK which is composed of the initial token $TK_{SN}^{I}$ and random numbers $n_{2}$ and $r_{2}$. Therefore, our proposed protocol supports mutual authentication between a sensor node and the gateway.
- *Forward Secrecy*: The aim of forward secrecy is to protect session keys generated in the past against compromises of session keys generated in the future. If the initial token $TK_{SN}^{I}$ is learned by an attacker who wants to derive the initial token $H\left(v⨁w⨁SK_{SN}\right)$ used in the previous session, the attacker needs to know the previously generated random numbers v and w. The random numbers v and w were generated by the sensor node and the gateway in the past authentication session. Since the attacker cannot obtain previously generated random numbers v and w from the previous eavesdropped messages, therefore, the attacker cannot use the current initial token $TK_{SN}^{I}$ to derive the pervious initial token. Hence, our proposed protocol has the property of forward secrecy.

In order to evaluate the security strength of the proposed protocol, an automatic verification tool for security protocols called *Scyther* [39] is used. *Scyther* adopts formal analysis methodology and assumes perfect cryptography, in which it is assumed that all cryptographic functions are perfect. An adversary learns nothing from an encrypted message unless he knows the decryption key. *Scyther* has been used to evaluate many practical protocols [39]. *Scyther* can analyze a target protocol for secrecy and authentication in terms of aliveness, weak agreement, non-injective agreement, and non-injective synchronization [40,41]. A security protocol definition language (spdl) is invented and used for *Scyther* to describe the details of the protocol which will be verified. Due to the syntax limitation of *Scyther*, we write two spdl scripts to describe two different conditions of the continuous authentication phase of the proposed protocol:

- Condition (1): The sensor has to relaunch the static authentication phase when the gateway identifies the value of ($t_{c}-t_{s}$) is larger than or equal to the valid authentication time period T. In this case, the gateway will construct special values of $Y_{1}$ and ACK. Once the sensor receives $Y_{1}$ and ACK from the gateway, it can determine whether relaunching the static authentication phase is required by evaluating the extracted value of $n_{2}^{\prime }$ from the received $Y_{1}$.
- Condition (2): Normal continuous authentication phase is executed.

**Figure 7:**
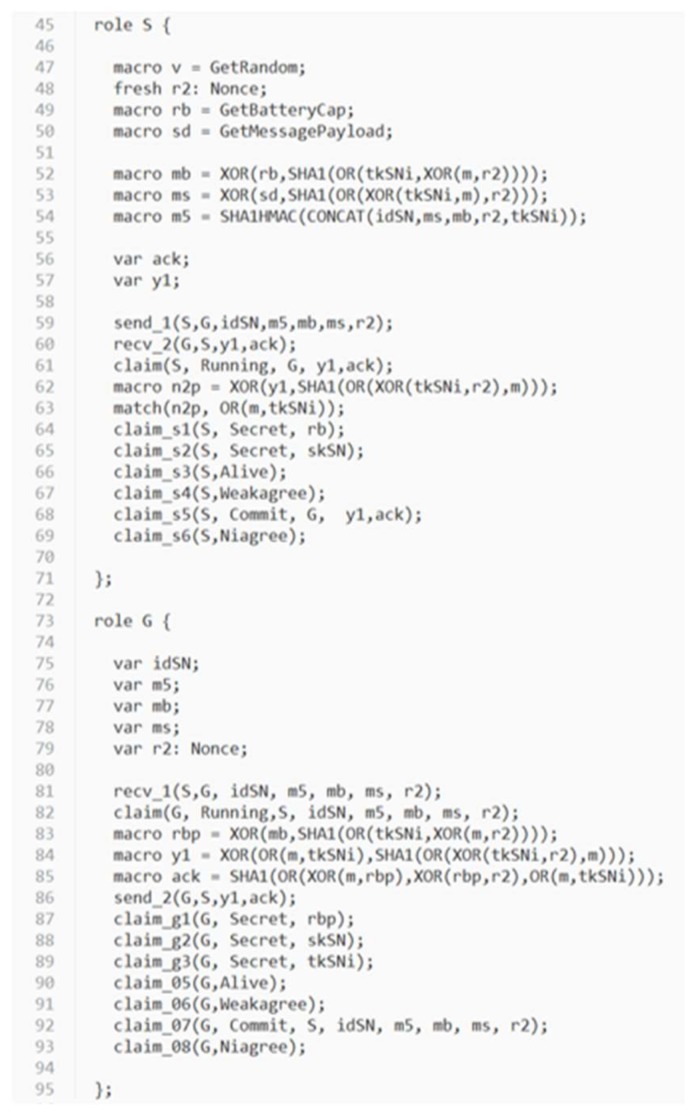
The spdl script for the static authentication phase of the proposed protocol.

**Figure 8:**
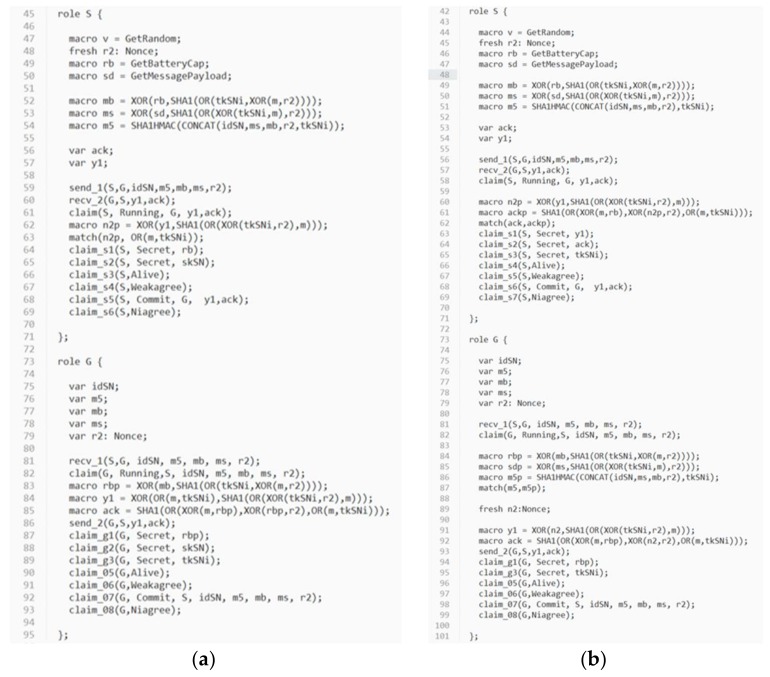
(**a**) The spdl script for the continuous authentication phase in Condition (1); (**b**) The spdl script for the continuous authentication phase in Condition (2).

**Figure 9:**
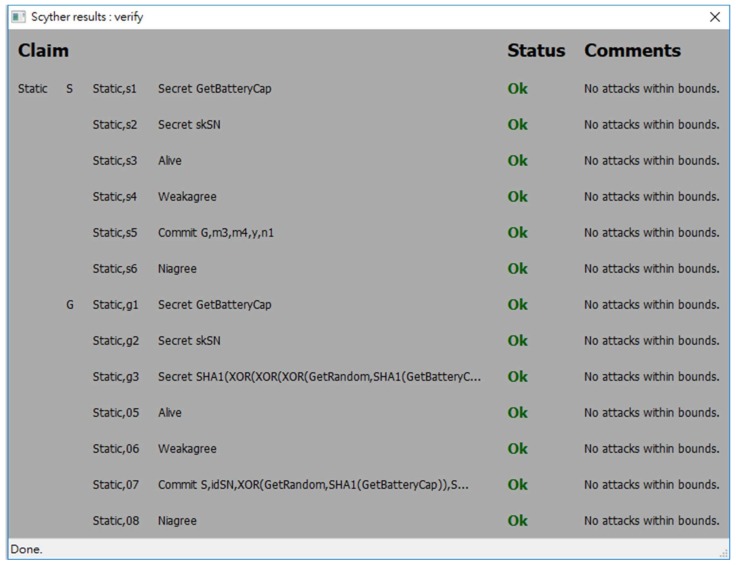
The security analysis result of the static authentication phase of the proposed protocol.

**Figure 10:**
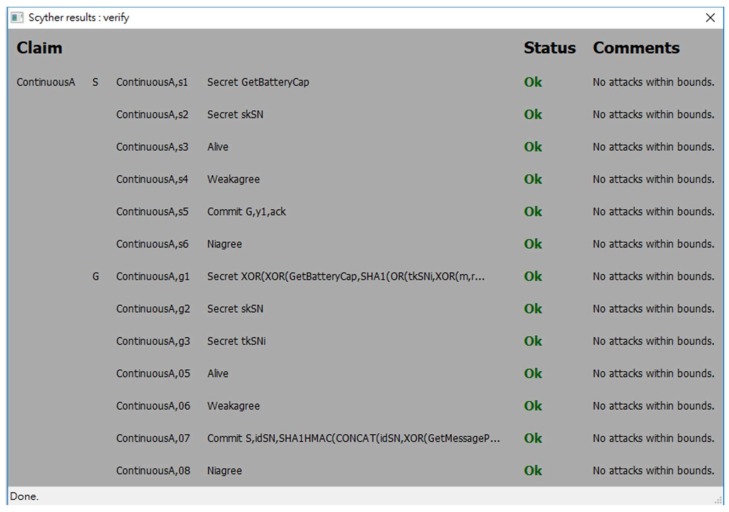
The security analysis result of the continuous authentication phase in Condition (1).

**Figure 11:**
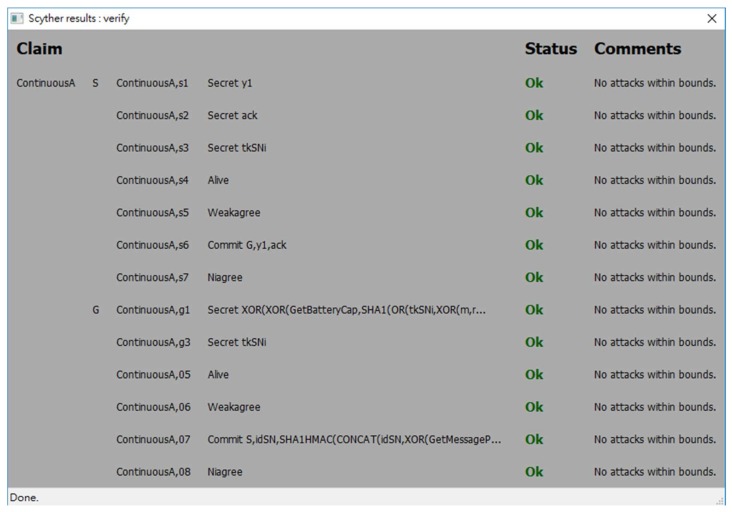
The security analysis result of the continuous authentication phase in Condition (2).

The spdl scripts for the static authentication phase and the continuous authentication phase of the proposed protocol are shown in Figure 7 and Figure 8. Figure 9, Figure 10 and Figure 11 show the execution results of our spdl scripts using *Scyther*. These results indicate no attacks are found within bounds, which were set as the maximum number of rounds. The term “no attacks within bounds” shown in Figure 9, Figure 10 and Figure 11 indicates that *Scyther* did not find any attacks by reaching the bound. The verification results prove that the proposed protocol is secure.

### 4.2. Performance Analysis

As mentioned in Section 2.2, we could not find other existing lightweight continuous authentication protocols to compare with the proposed protocol. Therefore, we planned to find a fast traditional user-to-device authentication protocol to compare with our protocol. In this subsection, we compare our proposed protocol with the protocol of Khemissa et al. [18] in terms of performance efficiency. The reason for selecting the protocol in [18] to compare with ours is that the protocol in [18] is a near lightweight authentication protocol. Therefore, its computation cost is already less than other existing authentication protocols we surveyed in Section 2.

Since the time consumption of executing a concatenation operation and bitwise exclusive-or operation is much less than other computing operations, we ignore time consumption generated by these two operations when calculating computation cost for an authentication protocol. As sensor nodes only equip limited computing resources and gateways usually have unlimited computing resources, we then focus on comparing the time consumption of the authentication process at the sensor node side between targeted protocols.

We define the following notations to indicate time consumption for different computing operations:

1. $T_{Hash}$: The consumed time of executing a hash function
2. $T_{AES}$: The consumed time of executing an AES operation
3. $T_{HMAC}$: The consumed time of executing a HMAC operation
4. $T_{Random}$: The consumed time of generating a random number.

Table 2 shows the comparison result of computation cost between the protocol of Khemissa et al. [18] and our proposed protocol. In the static authentication phase, our proposed protocol requires $4T_{Random}$ + 16$T_{Hash}$ + 4$T_{HMAC}$ while the protocol in [18] needs $2T_{Random}$ + $2T_{Hash}$ + 4$T_{HMAC}$ *+* $2T_{AES}$. In our proposed protocol, the lengths of $ID_{SN}$, random numbers ($r_{1},r_{2}$,$\;n_{1},n_{2},v,w$), and $SK_{sn}$ are all 128 bits. 

Based on the work in [42], the time consumption of AES encryption operation $T_{AES}$ is approximately 2.76 ms, the time consumption of hash operation $T_{Hash}$ is approximately 1.5 ms and the time consumption of HMAC operation $T_{HMAC}$ is approximately 3.54 ms. A pseudorandom number generation operation is approximately 0.65 ms as shown in [43]. Our protocol needs 12 extra hash function and two random number generation operations to complete static authentication in comparison with the protocol in [18]. On the other hand, our protocol reduces two AES encryption operation when comparing with the protocol in [18]. The continuous authentication phase of the proposed protocol only requires half of the computation operations of the static authentication phase. Notice that the time consumption between Condition (1) and Condition (2) has a slight difference as one hash operation using in Condition (1) is substituted by one HMAC operation in Condition (2).

In conclusion, the computation cost of the static authentication phase of the proposed protocol is around 40.76 ms while the computation cost of the protocol in [18] only takes 23.98 ms. However, the computation cost of the continuous authentication phase of the proposed protocol is around 18.34~20.38 ms. Notice that the computation cost of the protocol in [18] only counts for the authenticated session key generation. There will be extra computation cost when both communicating parties start to encrypt their transmitted messages using the agreed session key. In contrast, our protocol only generates the initial token agreed by the senor device and the gateway in the static authentication phase. Within the continuous authentication phase, sensed data along with the initial token and other temporary control values are transmitted from the sensor to the gateway. Therefore, our proposed protocol has better performance on continuous authentication in terms of computation cost. In summary, the proposed protocol is a very competitive protocol in terms of performance efficiency.

## 5. Discussion

In this section, two possible extensions of our proposed protocol are discussed: gateway initializing request and identity anonymity.

### 5.1. Gateway Initializing Request

**Figure 12:**
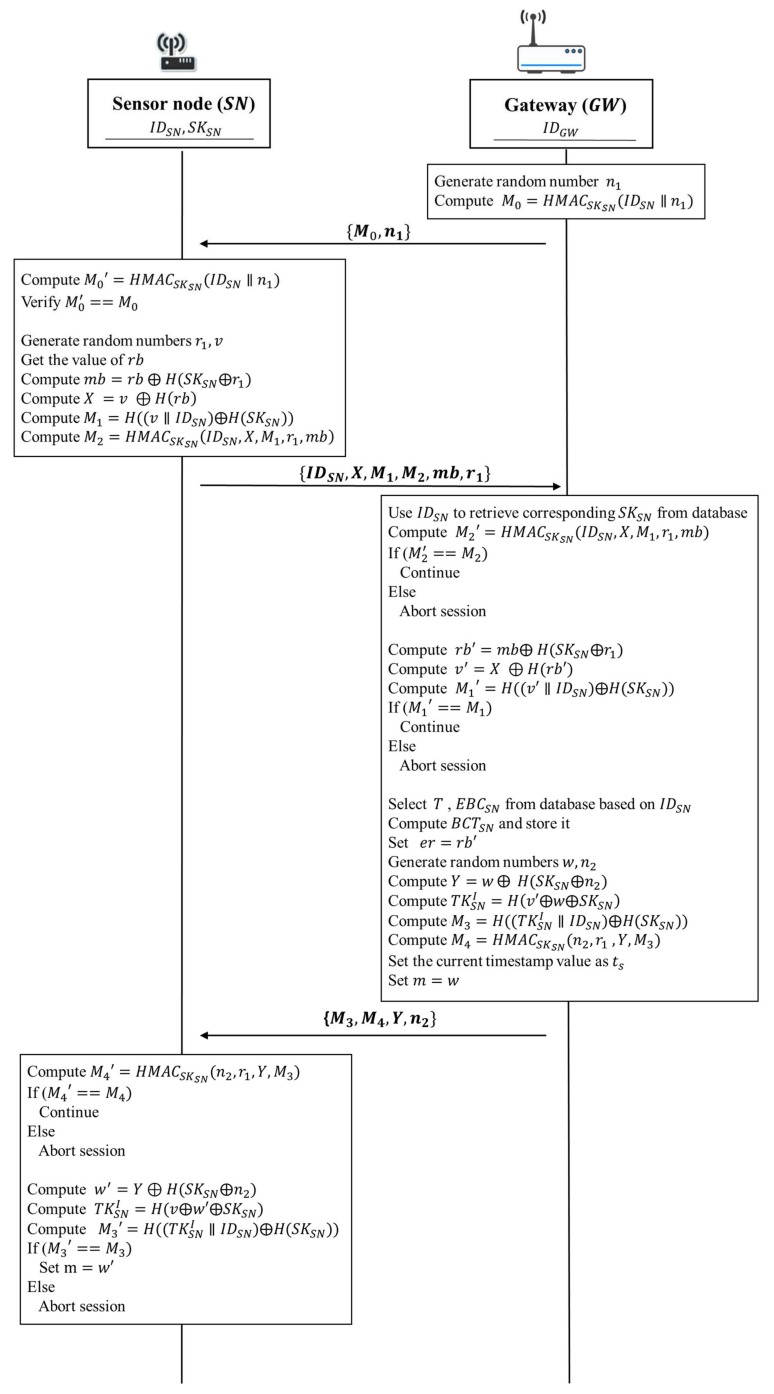
The static authentication phase for gateway initializing request.

In a general scenario, sensor nodes consecutively collect sensed data and send them to a gateway based on a scheduled time frame or the moment when the storage space of a sensor node is getting full. In special situations, the gateway may actively send a request to a targeted sensor node and ask for its sensed data. After receiving the request, the sensor node sends sensed data to the gateway immediately. By partially modifying our proposed protocol, our design can easily support the need for the gateway to initialize communication with a senor node. The static authentication phase of the modified protocol to support gateway initializing request is shown in Figure 12.

In the static authentication phase, the gateway generates a random number $n_{1}$ and computes $M_{0}=HMAC_{SK_{SN}}\left(ID_{SN}∥n_{1}\right)$ first. Then, the gateway sends $n_{1}$ and $M_{0}$ as a request to the targeted sensor node to ask for sensed data. After the sensor node receives $n_{1}$ and $M_{0}$, the sensor node uses the identity of the sensor node $ID_{SN}$ to compute $M_{0}\prime =HMAC_{SK_{SN}}\left(ID_{SN}∥n_{1}\right)$. If the computed value $M_{0}\prime$ and the received value $M_{0}$ are equivalent, the sensor node ensures the authenticity of the gateway. The rest of authenticating steps in the modified protocol are identical to the steps in the original proposed protocol. If necessary, the continuous authentication phase will be executed by both peers for the sensor node to keep transmitting sensed data to the gateway. The continuous authentication phase of the modified protocol is identical to the one in the original proposed protocol.

### 5.2. Identity Anonymity

**Figure 13:**
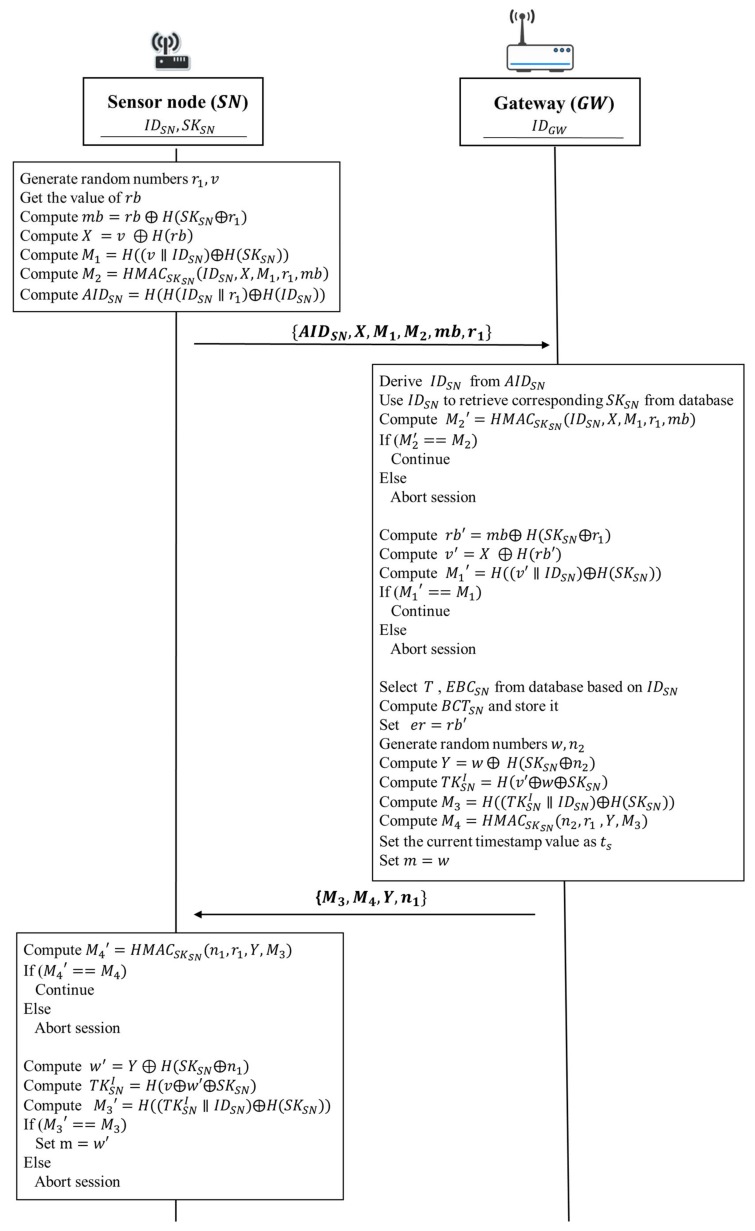
The static authentication phase with identity anonymity.

**Figure 14:**
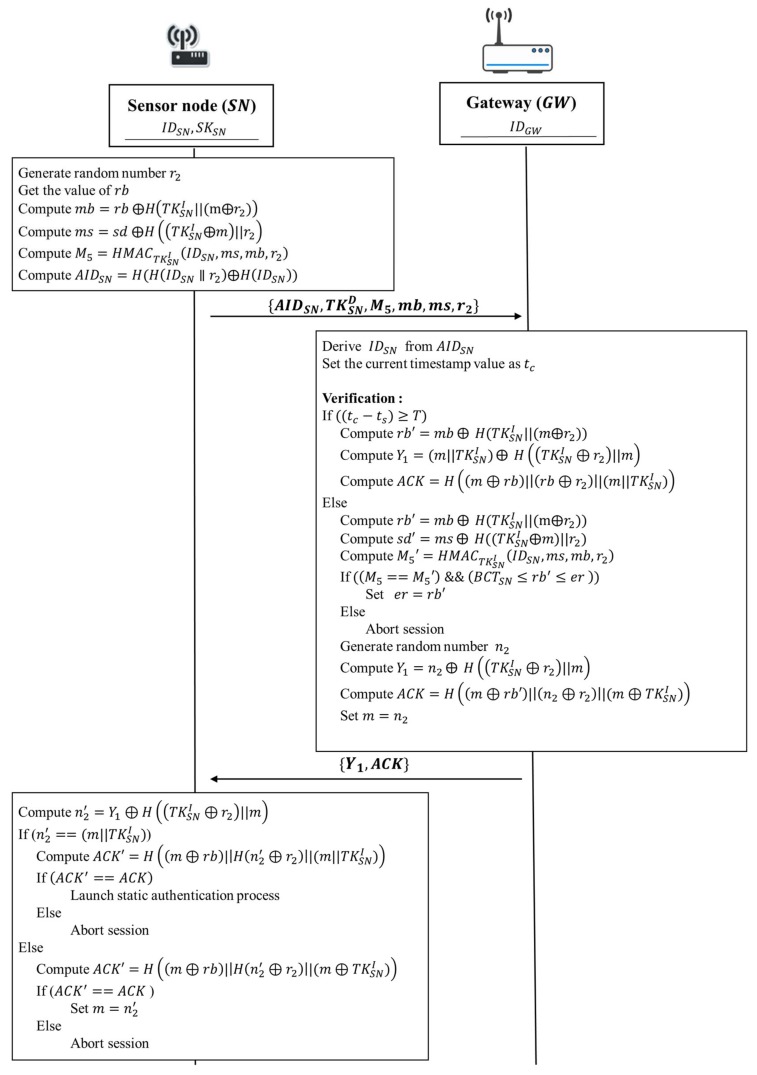
The continuous authentication phase with identity anonymity.

In some occasions, identity anonymity of a sensor node may be required. For the proposed protocol to achieve identity anonymity on sensor nodes, random number and hash functions are adopted to mask the actual identity of each sensor node and guarantee the uniqueness of each anonymous sensor identity $AID_{SN}$. A malicious attacker cannot derive the original identity of a sensor node $ID_{SN}$ from its anonymous identity $AID_{SN}$. The gateway needs additional computation time to determine the identity of a sensor node $ID_{SN}$ from a received $AID_{SN}$. The static authentication phase of our proposed protocol associated with the identity anonymity feature is shown in Figure 13 and the continuous authentication phase with the identity anonymity feature is shown in Figure 14.

In the static authentication phase and the continuous authentication phase of the modified protocol, the sensor node uses the random numbers $r_{1}$ and $r_{2}$ to compute an anonymous identity $AID_{SN}=H\left(H\left(ID_{SN}∥r_{1}\right)⨁H\left(ID_{SN}\right)\right)$ and $AID_{SN}=H\left(H\left(ID_{SN}∥r_{2}\right)⨁H\left(ID_{SN}\right)\right)$, respectively. In the modified protocol, the anonymous identity $AID_{SN}$ is used during data transmission instead of the original identity $ID_{SN}$. After the gateway receives $AID_{SN}$, it derives the identity of a sensor node $ID_{SN}$ by generating a tentative $AID\prime_{SN}=H\left(H\left(ID_{SN}∥r_{1}\right)⨁H\left(ID_{SN}\right)\right)$ for each sensor identity $ID_{SN}$ in the database and evaluating the equivalence between $AID\prime_{SN}$ and the received $AID_{SN}$.

## 6. Conclusions

In order to improve the social welfare of future human life through data analytics and the interaction interface between humans and their environment, new information systems with real-time sensed data from individuals and environments need to be developed and deployed in modern cities. To facilitate implementation of these systems, IoT-based devices such as sensing devices and intermediary gateways have to be deployed in different environments to form the IoT infrastructure. One of the fundamental security issues for IoT infrastructure is how to mutually authenticate both communicating peers before a sensing device transmits sensed data to an intermediary gateway. As sensed data will be transmitted to gateways periodically, a lightweight authentication protocol for device-to-device communication is indeed required to preserve device energy and extend the battery lifecycle correspondingly.

To accomplish this goal, a lightweight continuous authentication protocol for IoT infrastructure is proposed. The proposed protocol has several characteristics. First, the concept of valid authentication time period and continuous authentication are introduced and adopted. Second, the token technique and dynamic features of IoT devices, i.e., the remaining battery capacity, are adopted to quickly authenticate communicating parties in each session. Third, the computation process during an authentication session does not use encryption/decryption operations in order to reduce time consumption of these computation operations. In addition, a security analysis and a performance analysis are conducted for the proposed protocol to evaluate its security strength and its competitiveness in terms of time consumption for mutual authentication in a session. The proposed protocol can also be extended in two aspects: adopting the protocol implementation option of gateway-initialized request and adding the feature of identity anonymity onto sensing devices. For the future work, inventing a more accurate model of battery energy consumption and discovering more dynamic device features are two practical and interesting challenges for researchers.

## Figures and Tables

**Table 1:** The notations and their definitions.

Notation	Definition
SN	A sensor node
GW	A gateway
$ID_{SN}$	The identity of a sensor node SN
$ID_{GW}$	The identity of a gateway GW
$AID_{SN}$	The anonymous identity of a sensor node SN
T	The authentication period defined by the gateway for fast authenticating data transmission sessions after one successful static authentication. The time unit is by minute
$t_{s},\;t_{c}\;$	The timestamps
H(·)	A one-way hash function
$SK_{SN}$	The secret value of a sensor node SN
$r_{1},r_{2},v$	Random numbers generated by a sensor node SN
$n_{1},n_{2},\;w$	Random numbers generated by a gateway *GW*
$HMAC_{J}\left(\cdot \right)$	Hash-based message authentication code function associated with the secret key J
∥	A concatenation operation
⨁	A bitwise exclusive-or operation
sd	Sensed data from a sensor node SN
ms	The masked value of the sensed data from a sensor node SN
rb	The current energy capacity of sensor battery
er	The record of remaining energy capacity of sensor battery after last session
mb	The masked value of battery energy capacity
$TK_{SN}^{I}$	The initial token generated by a sensor node and the communicating gateway
$TK_{SN}^{D}$	The dynamic token generated by a sensor node
$EBC_{SN}$	The estimated daily average battery consumption value for a sensor node
$BCT_{SN}$	The estimated remaining battery capacity threshold for a sensor node to transmit data during a continuous authentication period T
ACK	Acknowledge message
$m,\;X,\;Y,Y_{1},\;M_{0},\;M_{1},\;$$M_{2},\;M_{3},M_{4},\;M_{5}$	Intermediate variables

**Table 2:** The comparison result on computation cost between the protocol of Khemissa et al. and the proposed protocol.

Phase	Khemissa et al. [15]	Our Protocol
Static authentication	$2T_{Random}$ + $2T_{Hash}\;$+ 4$T_{HMAC}$ *+* $2T_{AES}$	$4T_{Random}$ + 16$T_{Hash}$ + 4$T_{HMAC}$
Continuous authentication	--	Condition (1): 2$T_{Random}$ + 9$T_{Hash}$ + 1$T_{HMAC}$
		Condition (2): 2$T_{Random}$ + 8$T_{Hash}$ + 2$T_{HMAC}$

## References

[1] Perera C., Liu C.H., Jayawardena S. (2015). The Emerging Internet of Things Marketplace from an Industrial Perspective: A Survey. IEEE Trans. Emerg. Top. Comput..

[2] Coetzee L., Eksteen J. The Internet of Things—Promise for the Future? An Introduction. Proceedings of the IST-Africa Conference.

[3] Internet of Things (IoT) Cybersecurity Colloquium, National Institute of Standards and Technology (NIST), NISTIR 8201. Https://nvlpubs.nist.gov/nistpubs/ir/2017/NIST.IR.8201.pdf.

[4] AI-Fuqaha A., Guizani M., Mohammadi M., Aledhari M., Ayyash M. (2015). Internet of Things: A Survey on Enabling Technologies, Protocols, and Applications. IEEE Commun. Surv. Tutor..

[5] Mahmoud R., Yousuf T., Zualkeman I. Internet of things (IoT) Security: Current Status, Challenges and Prospective Measures. Proceedings of the 2015 10th International Conference for Internet Technology and Secured Transactions (ICITST).

[6] Shivraj V.L., Rajan M.A., Singh M., Balamuralidhar P. One Time Password Authentication Scheme Based on Elliptic Curves for Internet of Things (IoT). Proceedings of the 2015 5th National Symposium on Information Technology: Towards New Smart World (NSITNSW).

[7] Abomhara M., Køien G.M. Security and Privacy in the Internet of Things: Current Status and Open Issues. Proceedings of the 2014 International Conference on Privacy and Security in Mobile Systems (PRISMS).

[8] Alqassem I., Svetinovic D. A Taxonomy of Security and Privacy Requirements for the Internet of Things (IoT). Proceedings of the 2014 IEEE International Conference on Industrial Engineering and Engineering Management.

[9] Traore I., Woungang I., Nakkabi Y., Obaidat M.S., Ahmed A.A.E., Khalilian B. (2012). Dynamic Sample Size Detection in Learning Command Line Sequence for Continuous Authentication. IEEE Trans. Syst. Man Cybern..

[10] Mondal S., Bours P. Continuous Authentication in a Real World Settings. Proceedings of the 2015 Eighth International Conference on Advances in Pattern Recognition (ICAPR).

[11] Buduru A.B., Yau S.S. An Effective Approach to Continuous User Authentication for Touch Screen Smart Devices. Proceedings of the 2015 IEEE International Conference on Software Quality, Reliability and Security (QRS).

[12] Mondal S., Bours P. Continuous Authentication and Identification for Mobile Devices: Combining Security and Forensics. Proceedings of the 2015 IEEE International Workshop on Information Forensics and Security (WIFS).

[13] Brocardo M.L., Traore I., Woungang I. Toward a Framework for Continuous Authentication Using Stylometry. Proceedings of the 2014 IEEE 28th International Conference on Advanced Information Networking and Applications.

[14] Bamasag O.O., Youcef-Toumi K. Towards Continuous Authentication in Internet of Things Based on Secret Sharing Scheme. Proceedings of the WESS’15: Workshop on Embedded Systems Security.

[15] Bormann C., Ersue M., Keranen A. (2014). Terminology for Constrained-Node Networks. RFC 7228, Internet Engineering Task Force (IETF). https://tools.ietf.org/html/rfc7228.

[16] Sethi M., Arkko J., Keranen A., Back H. (2018). Practical Considerations and Implementation Experiences in Securing Smart Object Networks. Draft-Ietf-Lwig-Crypto-Sensors-06. https://tools.ietf.org/pdf/draft-ietf-lwig-crypto-sensors-06.pdf.

[17] Atzori L., Iera A., Morabito G., Giacomo M. (2010). The Internet of Things: A Survey. Comput. Netw..

[18] Khemissa H., Tandjaoui D. A Lightweight Authentication Scheme for E-Health Applications in the Context of Internet of Things. Proceedings of the 2015 9th International Conference on Next Generation Mobile Applications, Services and Technologies.

[19] Khemissa H., Tandjaoui D. A Novel Lightweight Authentication Scheme for Heterogeneous Wireless Sensor Networks in the Context of Internet of Things. Proceedings of the 2016 Wireless Telecommunications Symposium (WTS).

[20] Mahalle P.N., Prasad N.R., Prasad R. Threshold Cryptography-based Group Authentication (TCGA) Scheme for the Internet of Things (IoT). Proceedings of the 2014 4th International Conference on Wireless Communications, Vehicular Technology, Information Theory and Aerospace & Electronic Systems (VITAE).

[21] Porambage P., Schmitt C., Kumar P., Gurtov A., Ylianttila M. Two-phase Authentication Protocol for Wireless Sensor Networks in Distributed IoT Applications. Proceedings of the 2014 IEEE Wireless Communications and Networking Conference (WCNC).

[22] Krawczyk H., Bellare M., Canetti R. (1997). HMAC: Keyed-Hashing for Message Authentication. RFC 2104, Internet Engineering Task Force (IETF). https://www.rfc-editor.org/rfc/rfc2104.txt.

[23] Rescorla E., Modadugu N. (2012). Datagram Transport Layer Security Version 1.2. RFC 6347, Internet Engineering Task Force (IETF). https://www.rfc-editor.org/rfc/rfc6347.txt.

[24] Kothmayr T., Schmitt C., Hu W., Brünig M., Carle G. (2013). DTLS Based Security and Two-way Authentication for the Internet of Things. Ad Hoc Netw..

[25] Goh E.J. (2007). Encryption Schemes from Bilinear Maps. Ph.D. Thesis.

[26] Paillier P. (1999). Public-Key Cryptosystems Based on Composite Degree Residuosity Classes. Advances in Cryptology—EUROCRYPT ’99.

[27] Advanced Encryption Standard (AES), Federal Information Processing Standards Publication 197, National Institute of Standards and Technology (NIST). http://nvlpubs.nist.gov/nistpubs/FIPS/NIST.FIPS.197.pdf.

[28] Kumar P., Gurtov A., Iinatti J., Ylianttila M., Sain M. (2016). Lightweight and Secure Session-Key Establishment Scheme in Smart Home Environments. IEEE Sens. J..

[29] Gope P., Hwang T. (2015). Untraceable Sensor Movement in Distributed IoT Infrastructure. IEEE Sens. J..

[30] Kawamoto Y., Nishiyama H., Kato N., Shimizu Y., Takahara A., Jiang T. (2015). Effectively Collecting Data for the Location-Based Authentication in Internet of Things. IEEE Syst. J..

[31] Shimshon T., Moskovitch R., Rokach L., Elovici Y. Continuous Verification Using Keystroke Dynamics. Proceedings of the 2010 International Conference on Computational Intelligence and Security (CIS).

[32] Shen C., Cai Z., Guan X. Continuous Authentication for Mouse Dynamics: A Pattern-growth Approach. Proceedings of the IEEE/IFIP International Conference on Dependable Systems and Networks (DSN 2012).

[33] Bailey K.O., Okolica J.S., Peterson G.L. (2014). User Identification and Authentication Using Multi-modal Behavioral Biometrics. Comput. Secur..

[34] Niinuma K., Park U., Jain A.K. (2010). Soft Biometric Traits for Continuous User Authentication. IEEE Trans. Inf. Forensics Secur..

[35] Mock K., Hoanca B., Weaver J., Milton M. Real-time Continuous Iris Recognition for Authentication Using an Eye Tracker. Proceedings of the 2012 ACM Conference on Computer and Communications Security.

[36] Peng G., Zhou G., Nguyen D.T., Qi X., Yang Q., Wang S. (2017). Continuous Authentication with Touch Behavioral Biometrics and Voice on Wearable Glasses. IEEE Trans. Hum. Mach. Syst..

[37] Zhou L., Su C., Chiu W., Yeh K.H. (2017). You Think, Therefore You Are: Transparent Authentication System with Brainwave-oriented Bio-features for IoT Networks. IEEE Trans. Emerg. Top. Comput..

[38] Seitz L., Gerdes S., Selander G., Mani M., Kumar S. (2016). Use Cases for Authentication and Authorization in Constrained Environments. RFC 7744, Internet Engineering Task Force (IETF). https://tools.ietf.org/html/rfc7744.

[39] Scyther. https://www.cs.ox.ac.uk/people/cas.cremers/scyther/.

[40] Gavin L. A Hierarchy of Authentication Specifications. Proceedings of the 10th IEEE Workshop on Computer Security Foundations.

[41] Cremers C.J.F., Mauw S., Vink E.P. (2006). Injective Synchronisation: An Extension of the Authentication Hierarchy. Theor. Comput. Sci..

[42] Pereira G.C.C.F., Alves R.C.A., da Silva F.L., Azevedo R.M., Albertini B.C., Margi C.B. (2017). Performance Evaluation of Cryptographic Algorithms over IoT Platforms and Operating Systems. Secur. Commun. Netw..

[43] Yeh K.H., Su C., Choo K.R., Chiu W. (2017). A Novel Certificateless Signature Scheme for Smart Objects in the Internet-of-Things. Sensors.

